# Validity and Reliability of the Turkish Version of the Mindful Eating Behavior Scale

**DOI:** 10.3390/nu17193083

**Published:** 2025-09-28

**Authors:** Özge Dinç, Emine Akal Yıldız, Gözde Okburan, Zehra Buyuktuncer

**Affiliations:** 1 Faculty of Health Sciences, Nutrition and Dietetics, Eastern Mediterranean University, Famagusta 99628, Cyprus; emine.yildiz@emu.edu.tr (E.A.Y.); gozde.okburan@emu.edu.tr (G.O.); 2 Faculty of Health Sciences, Nutrition and Dietetics, Hacettepe University, Ankara 06100, Türkiye; zbtuncer@hacettepe.edu.tr

**Keywords:** mindful eating, eating behavior, eating awareness, validity and reliability

## Abstract

**Objectives**: The aim of this study was to evaluate the validity and reliability of the Mindful Eating Behavior Scale (MEBS) by Winkens et al. in Turkish adults. **Methods**: This cross-sectional validation study was conducted in three stages with 397 participants aged 18–65 years from Istanbul, Türkiye. Stage 1 involved linguistic validation (*n* = 50), Stage 2 included a psychometric evaluation (*n* = 397), and Stage 3 a test-retest reliability assessment (*n* = 50). Internal consistency was assessed using Cronbach’s alpha, and factor structure was examined through exploratory factor analysis (EFA), followed by confirmatory factor analysis (CFA). **Results**: The Turkish MEBS demonstrated excellent internal consistency (Cronbach’s *α* = 0.855) and good temporal stability (test-retest *r* = 0.821). EFA confirmed a four-factor structure explaining 64.01% of the total variance, consistent with the original validation. Factor loadings ranged from 0.50 to 0.85. CFA confirmed acceptable model fit (*χ*^2^*/df* = 3.147, CFI = 0.918, GFI = 0.910, RMSEA = 0.074). The Kaiser–Meyer–Olkin coefficient was 0.85, and Bartlett’s test was significant (*χ*^2^ = 2960.80, *p* < 0.05). **Conclusions**: The Turkish version of the MEBS demonstrates preliminary validity and reliability for assessing mindful eating behaviors in Turkish adults. This validation provides the first culturally adapted mindful eating assessment tool for Turkish clinical practice and research settings.

## 1. Introduction

The role of mindful eating, as a new concept in improving eating habits, is an emerging research interest of increasing importance [1]. Obesity and overweight are significant public health concerns. When the term “mindfulness” is used in the context of eating behaviors, it can be described as “Mindful Eating” or “Eating Awareness.” Mindful eating is defined as “focusing on the food to be consumed in the present moment, without judgment, being aware of how and why eating behavior occurs, internalizing the concepts of physical hunger and fullness, and recognizing the influence of emotions and thoughts without being affected by environmental factors.” Mindful eating can promote healthy living and eating habits, potentially supporting weight loss [2].

Mindfulness-based approaches are considered as the most effective method in addressing overeating, emotional eating, and eating in response to external cues [1]. Mindfulness-based interventions are implemented to promote behaviors related to maintaining an ideal body weight, reducing overweight or obesity, and enhancing adherence to adequate and balanced nutrition, as well as physical activity. Mindful awareness interventions related to eating can minimize automatic and impulsive reactions, thereby fostering self-regulation [3,4].

Reviews of mindfulness-based interventions focusing specifically on eating behaviors demonstrate their effectiveness in treating binge eating [5,6], weight management [6,7], emotional [6] and external eating [8], restricted eating and internal awareness [9], food consumption [8], and eating disorders [10]. These interventions have also been shown to reduce symptoms of depression and/or anxiety [11] and enhance mental well-being [12].

Mindfulness-based approaches may prevent weight gain. The potential mechanisms through which mindful eating is effective in obesity are the reduction in food cravings, a decrease in emotional eating behaviors, an improvement in meal nutritional quality, and the development of self-compassion, all of which appear to play a significant role in weight management. Several studies conducted on overweight or obese populations have shown that mindful eating can reduce food intake and that increased awareness in the specific domain of eating behaviors may contribute to the treatment of weight-related and disordered eating health issues [8,10,13]. Mindfulness and mindful eating have the potential to address problematic eating behaviors and the challenges many people face in controlling their food intake. Implementing the mindful eating approach represents a positive step toward integrating it into general weight management recommendations for the population [1,2].

Mindfulness-based interventions are increasingly used as a phenomenon to address eating behavior-related issues, ranging from eating disorders to weight management [10,14]. Mindfulness can also enhance long-term adherence to the lifestyle changes necessary for weight loss by promoting tolerance of the negative discomforts associated with calorie restrictions and increased physical activity [15]. The body’s biological stress response is linked to increased hunger, a preference for high-fat and high-sugar foods, and fat accumulation in the abdominal region. Thus, mindfulness meditation is proposed as a potentially beneficial intervention for individuals attempting to lose weight [16].

Excessive body weight represents one of the most challenging contemporary health issues in society. Problematic food and eating behaviors, such as overeating and emotional eating, are frequently associated with obesity [17]. Individuals with high levels of mindfulness observe their present-moment experiences with a clear, non-judgmental attitude. This awareness and acceptance of momentary experiences enables individuals to replace automatic thoughts and reactions with conscious and healthier responses [6,18].

Recent studies indicate that mindfulness-based approaches can prevent weight gain. Research conducted on overweight and obese populations demonstrates that such approaches can reduce food intake, although this effect is less pronounced in normal-weight populations [14,15,16]. Evidence suggests that mindful eating behavior is lower in overweight and obese individuals compared to those of normal weight. Increasing mindful eating is crucial for preventing obesity-related diseases and promoting a healthy lifestyle. Mindful eating enhances sensitivity to physical signs of hunger, fullness cues, and eating speed [19,20]. The mindful eating approach is considered a valuable component that can be integrated into weight management processes by increasing awareness of food and eating behaviors [19,21]. Given the potential contributions of mindful eating in preventing obesity and improving eating behaviors, assessing levels of mindful eating is essential. Consequently, validated measurement instruments for this domain are critically important.

Several validated questionnaires have been developed to assess conscious eating behavior, with the Mindful Eating Questionnaire (MEQ) by Framson et al. (2009) and Mindful Eating Scale (MES) serving as pioneering tools [22,23]. While the MEQ, a 28-item scale with five factors (distraction, external cues, emotional response, awareness, and disinhibition), was later abbreviated for time-constrained settings [24], its situation-specific items (e.g., restaurants, parties) limit its general applicability [22]. The MES was developed to include nonjudgment assessment and align with standard mindfulness definitions; however, while both scales measure eating awareness, they do not fully incorporate eating behavior itself, and the MES’s validation is limited by its predominantly female student sample [23].

The MEBS was designed as a new measurement tool for mindful eating, focusing on the attention component rather than emotional and external eating factors [25]. Unlike previous scales that combine mindful eating with emotional and external eating constructs, the MEBS uniquely isolates the attention component of mindful eating, enabling more precise measurement of mindfulness-specific eating behaviors. Additionally, its shorter length (17 items vs. 28 items in MEQ) makes it more practical for clinical assessments, while maintaining comprehensive coverage of mindful eating domains. Based on Brown and Ryan’s concept, the scale defines mindful eating as “Eating with attention and awareness,” incorporating two key components: self-regulation of attention in the present moment and attention given without judgment [26,27].

Cultural adaptation of mindful eating assessment tools is particularly important given that eating behaviors are deeply influenced by cultural norms, social contexts, and traditional food practices. In Türkiye, the increasing prevalence of obesity and eating disorders, combined with the lack of culturally validated mindful eating assessment instruments, creates a significant gap in research and limits the effectiveness of clinical practice [7]. Validating mindful eating scales across different populations is essential for understanding how cultural differences influence eating behaviors. Therefore, this study aimed to evaluate the validity and reliability of the Mindful Eating Behavior Scale (MEBS), developed by Winkens et al. [25], within the Turkish population. The comprehensive validation approach included both linguistic adaptation (translation, back-translation, and cultural adaptation) and psychometric evaluation (factor structure confirmation, internal consistency, and temporal stability assessment) to ensure the instrument’s appropriateness for the Turkish cultural context. This study intends to contribute a reliable tool to the literature that enhances knowledge regarding mindful eating in the Turkish population and supports adequate and balanced eating behaviors.

## 2. Materials and Methods

### 2.1. Study Design and Participants

This cross-sectional study was conducted in three stages between September 2023 and December 2023 with participants aged 18–65 (*X* ± *SD*: 28.8 ± 9.71 years) living in Istanbul, a metropolitan city in Türkiye. The participants were selected using the snowball sampling method. This comprehensive validation employed three stages: Stage 1 focused on linguistic adaptation and cultural appropriateness assessment; Stage 2 included a psychometric evaluation, including factor structure analysis and internal consistency testing; and Stage 3 involved pilot testing and temporal stability assessment through test-retest methodology. Each stage systematically established the instrument’s validity and reliability among Turkish adults. The snowball sampling method was chosen due to the specialized nature of mindful eating research, which required reaching participants with diverse demographic characteristics and varying levels of awareness regarding mindful eating concepts. This approach enabled access to hard-to-reach populations and facilitated the recruitment of participants across various socioeconomic and educational backgrounds in Istanbul’s metropolitan area. Individuals with any chronic illnesses requiring a specific diet and/or psychological disorders, those with eating disorders, pregnant and/or lactating individuals, or those who did not consent to participate were excluded from the study. Data collection was conducted through online surveys distributed via social media platforms and email networks using snowball sampling methodology. Participants completed the questionnaires digitally using a secure online survey platform, ensuring participant convenience and data standardization across all three study stages. The inclusion and exclusion criteria were applied consistently across all three stages of the research.

In the first phase, linguistic validation of the MEBS instrument—initially developed by Winkens et al. [25]—was performed with fifty participants.

**Figure 1:**
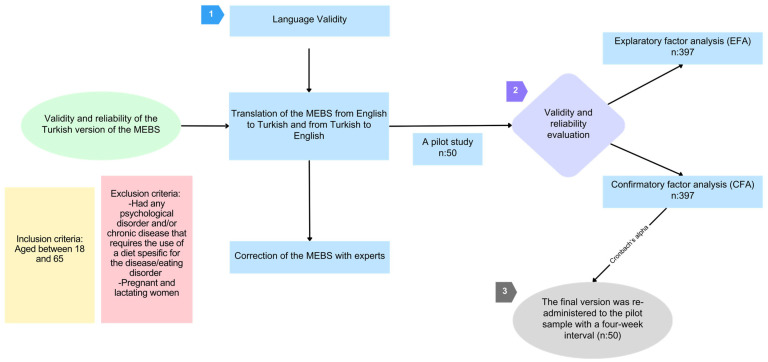
Flowchart of the study.

In the second phase, the sample size was determined through power analysis for an unknown population size with a 95% confidence interval and a 5% margin of error, indicating a minimum requirement of 384 participants [28,29]. The final sample of 397 participants exceeded this requirement and satisfied the recommendation for EFA of 5–10 times the number of items, ensuring adequate statistical power for factor analysis [30]. The research methodology is presented in Figure 1.

In the third phase, the scale obtained from the first two phases was administered to a pilot group to evaluate its effectiveness. The pilot study included 50 individuals. During this phase, participants provided feedback regarding item clarity, response format appropriateness, and questionnaire completion time. The overall feedback indicated satisfactory comprehension across all items and appropriate questionnaire length. Minor linguistic clarifications were requested for two items, which were addressed through minimal semantic adjustments while preserving equivalence with the original scale. Specifically, Item 11: “farkında olmadan” was refined to “fark etmeden” for improved clarity, and Item 15: “düşünürüm” was adjusted to “aklıma gelir” to better capture the intended meaning in the Turkish cultural context. The structural integrity of the instrument was maintained with no item additions or deletions. To assess the reliability of the MEBS, the final version was re-administered to the pilot sample (*n* = 50) using the test-retest method with a four-week interval. The Cronbach’s *α* coefficient for the scale was found to be excellent at 0.855, and the intraclass correlation coefficient indicated good reliability at 0.821 (95% CI: 0.73–0.88), based on the test-retest method.

For the scale validation procedures, permission was obtained from the authors of the original scale via email. Ethical authorization was granted by Eastern Mediterranean University Ethics Committee (approved on 11 July 2023, reference 2023/010), and all procedures aligned with the principles of the Declaration of Helsinki.

### 2.2. Mindful Eating Behavior Scale (MEBS)

The MEBS was established by Winkens et al. [25] to assess mindful eating behaviors, specifically targeting the attention component in adults. The MEBS comprises 4 factors, 4 domains, and 17 items. The factors relate to focused eating, physiological appetite indicators, eating with awareness, and distraction-free eating.

The MEBS indicates good preliminary convergent validity and internal consistency reliability among adults. The scale is scored with reverse coding for items in two of the four domains. Higher scores in each domain indicate higher levels of mindful eating. Due to the low correlations between factors, calculating a total score that combines the four domains is not recommended. Items 11 to 17 should be reverse coded as follows: 1 = 5; 2 = 4; 3 = 3; 4 = 2; 5 = 1. The primary advantage of the MEBS is that it contains only 17 items and can measure mindful eating domains independently of emotional, external, and restrained eating styles.

Scoring and Administration: The MEBS uses a 5-point Likert scale for all items (1 = never, 2 = seldom, 3 = sometimes, 4 = often, 5 = very often). Items 1–10 are scored directly, while items 11–17 require reverse coding (1 = 5, 2 = 4, 3 = 3, 4 = 2, 5 = 1). The four subscales are composed as follows: Focused Eating (items 1–5), Hunger and Satiety Cues (items 6–10), Eating with Awareness (items 11–13), and Eating without Distraction (items 14–17). Each subscale should be scored separately, and total scale scores are not recommended due to low inter-factor correlations. Higher scores on each subscale indicate higher levels of mindful eating behaviors.

Missing Data Management: For missing data handling, participants with more than 20% missing responses within any subscale should be excluded from the analysis. For any remaining missing values, mean imputation at the item level within the respective subscale is recommended to maintain statistical power while preserving the integrity of the measurement.

### 2.3. Language Validity

The process of adapting the scale to the Turkish language initially focused on ensuring language validity. The scale was translated from the original language (English) to Turkish by academics proficient in the two languages, following the language validity procedures suggested by Brislin (1986) and Prieto (1992) [31,32,33].

As recommended by Bracken and Barona [34], all statements in the scale were revised through repeated back-translation comparisons until both versions were fully aligned. The standard back translation procedure was used to minimize differences in expression during cross-linguistic adaptation. The translated scale was back-translated by two independent academics fluent in English and not affiliated with the authors’ institutions. The resulting Turkish-adapted MEBS was examined by the researchers to ensure clarity in expressions and suitability for both the research and the cultural context. The back-translated versions were then compared with the original English version for inconsistencies, errors, biases, and discrepancies. Following the recommendations of Bracken and Barona (1991), these inconsistencies were resolved by repeating the back-translation comparison procedure until both versions were identical [34]. The initial translation from English to Turkish was performed independently by three qualified experts: a nutrition specialist with advanced English proficiency, a graduate of English Language and Literature, and an expert who had graduated from an international university providing English-medium instruction. The back-translation process was conducted by two independent academics proficient in both languages and unaffiliated with the authors’ institutions. The reconciliation process was performed by the research team to ensure translation accuracy and cultural adaptation.

In the next phase of the study, each translated item was independently examined by six experts to confirm whether it accurately reflected the intended purpose of the scale. Expert evaluations were assessed using the Content Validity Index (CVI). Item-Level Content Validity Index (I-CVI) for each item and Scale-Level Content Validity Index (S-CVI) for the entire scale were used to calculate the CVI.

The quantitative content validity results demonstrated excellent agreement among experts. The per-item I-CVI values ranged from 0.83 to 1.00, with all items meeting the minimum threshold of 0.83 required for six experts. Fifteen items achieved perfect agreement (I-CVI = 1.00), while two items received I-CVI = 0.83. The Scale-Level Content Validity Index using the average method (S-CVI/Ave) was 0.94, exceeding the recommended threshold of 0.90.

The researchers examined the scale for meaning and derived the Turkish text from the most appropriate expressions. After all statements were corrected by the researchers, the MEBS was administered to 50 individuals who met the inclusion criteria, and the scale was finalized based on their suggestions regarding clarity of meaning.

### 2.4. Reliability and Validity Evaluation

Following the language adaptation stage, the Cronbach’s α coefficient, one of the most frequently used methods for assessing internal consistency, was utilized to evaluate scale reliability [35]. As a measure of the internal consistency of scores, Cronbach’s *α* coefficients were calculated for each measurement [36]. High α coefficients indicate strong internal consistency of the scores [37]. According to standards outlined by Kline (2016), the Cronbach’s *α* coefficient required for reliability should be at least 0.70 [38].

The scale was evaluated for construct validity using EFA. Preliminary examination of the dataset verified that the necessary assumptions for applying EFA were met. In this research, the Kaiser–Meyer–Olkin (KMO) coefficient was implemented to assess data adequacy, and Bartlett’s test of sphericity was applied to determine whether correlations existed among the items, which is a prerequisite for factor analysis. The KMO coefficient is required to be ≥0.60, and the statistical outcome of Bartlett’s test of sphericity should be less than 0.05 [39]. To determine scale item necessity, analysis focused on the factor loadings obtained from exploratory factor analysis and corrected item-total score correlations.

After assessing the appropriateness of the scale for factor analysis, varimax rotation principal component analysis was employed to assess the theoretical validity and factor structure of the scale using EFA. Factors with an eigenvalue (*λ*) ≥ 1.0, derived through extraction of the principal components, were retained. Following recommended best practices for ordinal Likert data [40], supplementary factor analysis was conducted using Principal Axis Factoring (PAF) with Promax oblique rotation on polychoric correlation matrices to evaluate the robustness of the factor structure. This approach accounts for the ordinal nature of Likert responses and allows for correlated factors, providing a more theoretically appropriate analysis for latent construct validation.

Confirmatory factor analysis was employed to contrast the factor structure of the adapted MEBS with that of the original MEBS, aiming to identify similarities and differences and evaluate the model’s suitability for the relevant population.

The factor structure emerging from the EFA was re-examined with CFA without making any changes to the items, and chi-squared/degrees of freedom (*χ*^2^*/df*), root mean square error of approximation (RMSEA), SRMR (Standardized Root Mean Square Residual), comparative fit index (CFI), and the Tucker–Lewis index were reported as the fit indices.

### 2.5. Statistical Evaluation

Statistical analyses of the research data were conducted using IBM Statistical Package for Social Sciences (SPSS) 26 and IBM SPSS AMOS 21.0 software. For polychoric correlation matrices and Principal Axis Factoring with Promax rotation, SPSS 26.0 matrix procedures were employed. Test-retest reliability was assessed using the intraclass correlation coefficient (ICC) with 95% confidence intervals. For the validity analysis, the suitability of the dataset for factor analysis was first assessed through the KMO, Bartlett’s test of sphericity, and multivariate normality tests. After confirming these assumptions, EFA was conducted, followed by CFA. For the reliability study, the Cronbach’s alpha test, split-half test, and item-total correlations were examined. Factor loading thresholds of ≥0.30 were used for item retention decisions following the recommendations from Tabachnick et al. (2013) [40]. Cross-loadings > 0.30 on secondary factors were examined for interpretation. Confidence intervals (95% CI) were calculated for all reliability estimates to enhance interpretability.

## 3. Results

### 3.1. Study Population

To determine the reliability and validity of the scale, 397 inhabitants of Istanbul aged between 18 and 65 were recruited, based on a 95% confidence interval and a 5% sampling error. The mean age of these individuals was 28.8 ± 9.71 years, with 243 women (61.2%) and 154 men (38.8%) participating in the study. Demographic data collection was intentionally limited to essential variables (age and gender) to maximize response rates and minimize participant burden in the online survey format, while ensuring adequate sample size for robust psychometric evaluation.

### 3.2. Reliability and Validity Analysis

The dataset was preliminarily examined to confirm that the assumptions required for conducting EFA were met. In this context, the draft version of the Mindful Eating Behavior Scale for Adults was first analyzed for multivariate normality, which was confirmed to be acceptable. Additionally, the Kaiser–Meyer–Olkin (KMO) coefficient and Bartlett’s test of sphericity were used to assess suitability. The KMO coefficient for the scale’s factor analysis was calculated as 0.85; for factor analysis, the KMO value should be ≥0.60. The KMO result further indicated that the sample size was adequate. Bartlett’s test of sphericity examines relationships among variables based on partial correlations, assessing whether this relationship is statistically significant. The results of Bartlett’s test showed that the level of correlation among items was sufficient for factor analysis (*χ*^2^ = 2960.80; *p* < 0.05). Based on these results, the scale was evaluated for construct validity through EFA, following the method recommended by Tabachnick et al. (2013) [40].

Examination of the scree plot for determining the factor structure of the Mindful Eating Behavior Scale for Adults supports the hypothesis that the scale consists of four factors. EFA was conducted to determine the number of factors on which the 17 items of the scale are distributed and to reveal the scale’s factor structure.

The results of the exploratory factor analysis conducted on the Mindful Eating Behavior Scale for Adults are presented in Table 1. This analysis was performed using the Principal Component Analysis (PCA) method to determine the scale’s internal factor structure. PCA is a statistical approach used to understand relationships among variables in multivariate datasets and to reduce data dimensionality. This approach calculates correlations among variables in the dataset, grouping related variables to form new components [41].

The Mindful Eating Behavior Scale for Adults consists of four distinct factors, explaining 64.01% of the total variance (Table 1). Tabachnick et al. (2013) recommend a minimum factor loading of 0.30, and the factor loadings of the scale items range from 0.50 to 0.85 [40]. These results indicate that the factor loadings of the scale items are acceptable and that the original form maintains a four-factor structure. Exploratory factor analysis revealed a four-factor structure for the Turkish version, confirming the four-factor structure described for the original. This structural consistency demonstrates the cross-cultural validity of the MEBS factor structure across Dutch and Turkish populations.

To ensure the robustness of our factor structure, supplementary analysis using Principal Axis Factoring (PAF) with Promax oblique rotation was conducted on polychoric correlation matrices. Sample adequacy remained excellent (KMO = 0.848; Bartlett’s *χ*^2^ = 2895.42, *df* = 136, *p* < 0.001). PAF analysis confirmed the four-factor structure with eigenvalues of 3.41, 2.83, 2.65, and 2.25, explaining 65.6% of the total variance (compared to 64.0% in the original PCA). Factor loadings were strengthened: Factor 1 (Focused Eating, Items 1–5) showed pattern loadings ranging from 0.51 to 0.85; Factor 2 (Hunger and Satiety Cues, Items 6–10) ranged from 0.62 to 0.87; Factor 3 (Eating with Awareness, Items 11–13) ranged from 0.79 to 0.87; and Factor 4 (Eating without Distraction, Items 14–17) ranged from 0.76 to 0.83. Oblique rotation revealed low inter-factor correlations (*r* = 0.13–0.20), indicating that both orthogonal and oblique approaches are appropriate. Communalities ranged from 0.43 to 0.85, demonstrating adequate factor extraction. All 17 items maintained identical factor assignments, confirming cross-methodological structural validity.

CFA was applied to evaluate the relationships between factors and confirm the suitability of the factor structure for identifying similarities and differences, as well as assessing the model’s relevance to the target population. CFA is regarded as a statistical method that builds upon EFA [42].

Various fit indices are used to evaluate the results of CFA to determine how well the model aligns with the data. The items in the factor structure identified through EFA were re-examined using CFA without any modifications. The *χ*^2^*/df* (chi–squared/degrees of freedom) value was calculated as 3.147, indicating good model fit as values between 3 and 5 are considered acceptable [43]. The goodness of fit index (GFI), normed fit index (NFI), comparative fit index (CFI), and RMSEA (root mean square error of approximation) were also assessed. As recommended by Kline (2016), GFI, NFI, and CFI indices should exceed 0.90 to indicate acceptable model fit [38]. For the Mindful Eating Behavior Scale for Adults, the GFI was 0.910, the NFI was 0.905, and the CFI was 0.918, indicating good model fit. The RMSEA was calculated as 0.074. The SRMR was 0.069, indicating good model fit (criterion < 0.08). The TLI was 0.887, a value approaching the acceptable threshold of 0.90. Given the large sample size (*n* = 397) and 5-point Likert scale format, the use of Maximum Likelihood (ML) estimation was appropriate. Multivariate normality assumptions were assessed through examination of skewness and kurtosis values for each item. Standardized factor loadings, item residuals, and inter-factor correlations were calculated to provide comprehensive CFA model evaluation. According to Tucker and Lewis (1973), RMSEA values should be below 0.08 [44]. In this context, the RMSEA value for the Mindful Eating Behavior Scale for Adults was determined to be appropriate.

Additionally, Average Variance Extracted (AVE) values were calculated to assess convergent validity. The results for the four factors were as follows: Focused Eating (AVE = 0.39), Hunger and Satiety Cues (AVE = 0.61), Eating with Awareness (AVE = 0.65), and Eating without Distraction (AVE = 0.52). Three factors met the ≥0.50 threshold for adequate convergent validity, though the Focused Eating factor fell below this criterion (AVE = 0.39). However, this factor demonstrated adequate composite reliability (CR = 0.788) and all factor loadings exceeded 0.48, indicating acceptable convergent validity according to the Fornell–Larcker criterion where composite reliability > 0.60 can compensate for AVE < 0.50 [45].

**Figure 2:**
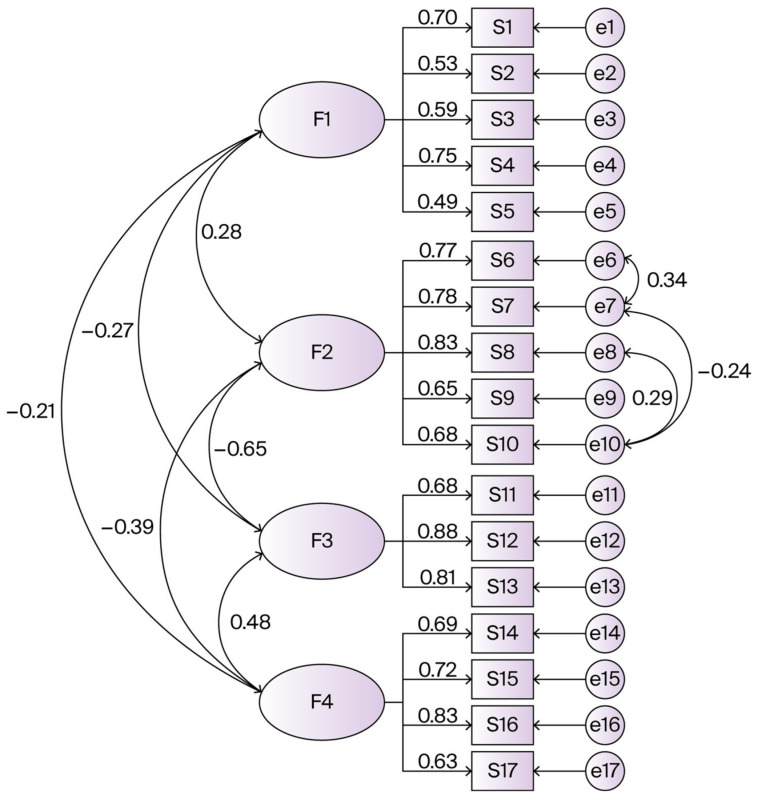
Path diagram of the confirmatory factor analysis (CFA) for the four-factor structure of the Mindful Eating Behavior Scale for Adults.

The items in the factor structure that emerged from EFA were re-examined with CFA without changing them, and the *χ*^2^*/df*, RMSEA, GFI, CFI and NFI fit indices were calculated, as shown in Table 2. According to these results, the CFA model for the Mindful Eating Behavior Scale for Adults is suitable for the data, and the factor structure is valid. This analysis supports the construct validity of the scale. Given these results, it can be concluded that all items in the scale are appropriate and that no items should be removed (Figure 2). According to the fit indices used in this study, the model demonstrates an acceptable fit. Based on these results, the values obtained from the scale confirm the acceptability and applicability of the Turkish version of the MEBS.

Following the adaptation of the scale to Turkish, internal consistency reliability was evaluated using multiple indices, with a focus on subscale-level assessment. The Cronbach’s alpha values for the four subscales were as follows: Focused Eating (*α* = 0.726, 95% CI: 0.67–0.78), Hunger and Satiety Cues (*α* = 0.865, 95% CI: 0.84–0.89), Eating with Awareness (α = 0.821, 95% CI: 0.78–0.86), and Eating without Distraction (*α* = 0.723, 95% CI: 0.66–0.78). All subscale reliabilities exceeded the minimum threshold of 0.70, demonstrating adequate internal consistency. For comprehensive reliability assessment, McDonald’s omega (*ω* = 0.86, 95% CI: 0.82–0.89) and composite reliability (CR = 0.87) were also calculated alongside the overall Cronbach’s alpha (*α* = 0.855, 95% CI: 0.81–0.88). The factor-specific composite reliability values were as follows: Focused Eating (CR = 0.788), Hunger and Satiety Cues (CR = 0.871), Eating with Awareness (CR = 0.834), and Eating without Distraction (CR = 0.847). All values exceeded the 0.70 threshold for adequate reliability. These results demonstrate that the scale has internal consistency and can produce reliable results. These findings support the reliability of results obtained in studies using the MEBS (Table 3).

The correlations between the items in the Mindful Eating Behavior Scale for Adults and the total score were examined. The correlation coefficients ranged from 0.258 to 0.659. The highest correlation was found with item eight, “I trust my body to tell me how much to eat,” while the lowest correlation was observed with item 4, “I notice the smells and aromas of food.”

The split-half reliability variables for the Mindful Eating Behavior Scale for Adults demonstrated alpha values of 0.792 for the first half and 0.793 for the second half. The correlation coefficient between the first and second halves was 0.548. Additionally, the Spearman–Brown correlation coefficient was calculated as 0.708, and the Guttman split-half correlation coefficient was 0.709. These data support the consistency of the items within the scale and confirm that the scale provides reliable results.

The results of the analysis reveal that the Turkish version of the Mindful Eating Behavior Scale is valid and reliable with four factors and seventeen items. This finding indicates that the relationships between factors and the structure of the scale are valid and capable of producing reliable results.

Table 4 presents detailed CFA results, including standardized factor loadings and item-level parameters. All factor loadings exceeded 0.48 and were statistically significant (*p* < 0.001). Item residuals ranged from 0.30 to 0.77, with *R*^2^ values indicating that factors explained 24–70% of item variance. Inter-factor correlations were moderate, ranging from 0.20 to 0.56, with the highest correlation between Factors 2 and 3 (*r* = 0.562). Assessment of multivariate normality revealed departures from normality typical of Likert scale data, with most items showing negative skewness. The TLI value (0.887) approached but did not reach the conventional 0.90 threshold.

## 4. Discussion

This study aimed to evaluate the validity and reliability of the Turkish version of the Mindful Eating Behavior Scale (MEBS) through comprehensive linguistic adaptation and psychometric evaluation. The validation process successfully demonstrated that the Turkish MEBS is a psychometrically sound instrument with excellent internal consistency (Cronbach’s *α* = 0.855) and good temporal stability (test-retest *r* = 0.821). Exploratory factor analysis revealed a four-factor structure explaining 64.01% of the total variance, consistent with the original four-factor structure, confirming the structural validity of the MEBS in the Turkish population.

The validation of the MEBS for the Turkish population addresses a critical gap in culturally appropriate assessment tools. Despite the rising prevalence of obesity and eating disorders in Türkiye, no validated mindful eating assessment instruments exist for use in Turkish clinical practice and research settings [13]. The replication of the original four-factor structure in this Istanbul-based sample provides preliminary evidence for the cross-cultural validity of the MEBS construct; however, additional research across diverse Turkish populations and geographical regions would strengthen conclusions regarding the instrument’s broader applicability within this cultural context. This finding provides preliminary support for the cross-cultural applicability of the MEBS, pending validation in wider and more representative Turkish samples.

In contrast to previous scales, the MEBS enables the differentiation of mindful eating from other eating behaviors that may have varying effects on health outcomes by specifically focusing on the attention component of mindful eating and excluding items related to emotional and external eating. This approach, which emphasizes pure attention and awareness during eating, makes it possible to more precisely examine mechanisms in research studies. Furthermore, it is beneficial for applied settings, as it enables better adaptation of treatment to an individual’s needs [46].

According to the results of this study, the Turkish version of the Mindful Eating Behavior Scale, which consists of 17 items and four domains—including Focused Eating, Hunger and Satiety Cues, Eating with Awareness, and Eating without Distraction—is valid and reliable. From the EFA results, all item loadings were determined to be acceptable, ranging from 0.50 to 0.85, which exceeds the minimum threshold of 0.30 recommended by Tabachnick et al. (2013) [40]. In this study, the item-total correlation coefficients were found to range between 0.258 and 0.659. The values for Cronbach’s α internal reliability coefficients were strong and similar to the original scale. The reliability coefficients of each subscale and the overall scale were found to be above 0.70. Among the Cronbach’s *α* values obtained for the subscales, the lowest Cronbach’s *α* coefficient was 0.723 for the Eating without Distraction subscale. These reliability differences may be attributed to demographic characteristics of our sample, particularly the younger age (mean = 28.8 years) and gender composition compared to the original Dutch validation study’s older adult cohort (55+ years), as research suggests that age and gender can influence response patterns in psychometric assessments of eating behaviors. In the original study, Cronbach’s α was 0.80 for the three subscales of Focused Eating, Hunger and Satiety Cues, and Eating with Awareness, while it was 0.70 for the Eating without Distraction subscale. Similar to this study, the original study also achieved the high values desired when evaluating *χ*^2^*/df*, CFI, and RMSEA values. The four-factor structure found in this study aligns with the original four-factor Dutch version, confirming the cross-cultural validity of the MEBS structure. The high internal consistency coefficients across all factors (ranging from 0.723 to 0.865) demonstrate coherent response patterns within each mindful eating domain, indicating that these constructs are meaningful and well-understood across different populations. The particularly strong reliability in “Hunger and Satiety Cues” (*α* = 0.865) exceeds the original study’s reliability coefficients for this domain. While the original study reported Cronbach’s α values ranging from 0.70 to 0.80 across subscales, the Turkish version demonstrated higher internal consistency (0.723–0.865), indicating excellent psychometric performance. The higher reliability coefficients in our Turkish sample may be attributed to specific characteristics of our sample, as our participants were younger (mean age 28.8 years) and included both genders, compared to the original Dutch validation study with participants aged 55+ years. Younger populations may demonstrate more consistent response patterns to mindful eating items. This pattern of structural consistency with strong psychometric performance supports the cross-cultural validity of mindful eating instruments and demonstrates the successful adaptation of the MEBS for Turkish populations. The model fit indices demonstrated acceptable values that were similar to those found for the original scale [25]. The detailed CFA revealed moderate inter-factor correlations (*r* = 0.20–0.56), supporting the theoretical relationships between mindful eating constructs while maintaining factorial validity. The TLI value was marginally below the 0.90 threshold, likely reflecting the ordinal nature of Likert data and the associated normality violations common in psychometric research. Maximum Likelihood estimation was employed given the large sample size; however, future studies should consider robust estimation methods (WLSMV) specifically designed for ordinal data to optimize model fit indices. The structural validity of the Turkish MEBS was further supported by supplementary Principal Axis Factoring analysis, which confirmed the four-factor solution with enhanced factor loadings and equivalent variance explanation. The consistency of results across both PCA and PAF approaches demonstrates the robustness of the factor structure and supports the cross-cultural applicability of the MEBS construct in Turkish populations.

The Turkish MEBS fills an important gap in Turkish nutrition research by providing a validated instrument for assessing mindful eating behaviors. The MEBS differs from other eating behavior scales by focusing specifically on the attention component of mindful eating, rather than combining it with emotional and external eating factors [25]. This approach allows researchers to examine mindful eating mechanisms more precisely in Turkish populations. Given the increasing use of mindfulness-based interventions for eating-related problems, from obesity to eating disorders, the availability of a validated Turkish MEBS is particularly relevant for clinical practice and research [47,48,49]. The excellent psychometric properties observed in this study demonstrate that the adapted scale can provide reliable measurements in Turkish settings. The 17-item format is also practical for use in large-scale studies and clinical assessments.

The Turkish MEBS results align with research demonstrating the importance of attention during eating. Previous studies have shown that focusing on sensory characteristics during eating leads to greater food satisfaction and lower intake [48]. This supports the relevance of the “Focused Eating” factor, which showed the highest factor loading in our validation, indicating that Turkish participants effectively recognize attention to food characteristics. Conversely, the “Eating without Distraction” factor showed the lowest mean scores in our sample, suggesting areas for improvement that align with research showing distraction increases food consumption [48]. Analysis of the MEBS subscale mean values revealed that the Focused Eating factor, comprising food and eating situation items, was associated with the highest value. These findings align with other studies reporting focused eating as a significant criterion for mindful eating [50,51]. Environmental distractors can influence the amount of food consumed, food choice, hunger-satiety perception, and food enjoyment [52]. Research confirms that various forms of distraction negatively impact eating awareness, supporting the importance of the “Eating without Distraction” factor in the MEBS [53,54,55].

Recent studies have demonstrated a causal relationship between distraction and increased food consumption (both immediately and subsequently), highlighting the need for a better understanding of how distraction-related food intake relates to body weight [56]. Eating mindfully reduces food cravings and facilitates weight control, thus playing an important role in weight management [57]. The “Eating without Distraction” factor of the MEBS can be utilized in mindfulness-based intervention studies to identify the impact of environmental distractors on eating behavior, potentially contributing to improved eating awareness by enabling distracting factors to be eliminated.

The psychometric properties of the Turkish MEBS suggest potential applications across different professional domains—pending further validation in specific clinical contexts. In clinical dietetics practice, the scale can be used to assess baseline mindful eating levels before implementing nutrition interventions and monitor progress throughout treatment. For mental health professionals, the MEBS can serve as a screening tool to identify individuals who may benefit from mindfulness-based eating interventions, particularly those with emotional eating patterns or disordered eating behaviors. In public health settings, the scale could be integrated into community-based obesity prevention programs to evaluate intervention effectiveness. Additionally, the brief 17-item format makes the MEBS suitable for digital health platforms and mobile applications focused on mindful eating training. Research applications include pre-post intervention studies, cross-sectional population assessments, and as an outcome measure in randomized controlled trials examining mindfulness-based eating interventions in Turkish populations.

### Strengths and Limitations

The strengths of this study include the high reliability coefficients (Cronbach’s *α* = 0.855, test-retest = 0.821), a robust factorial structure confirmed by both EFA and CFA, and systematic cultural adaptation using the Brislin Procedure in the translation process.

Limitations include the cross-sectional study design, which constrains assessment of long-term stability in mindful eating behaviors. The absence of convergent and discriminant validity testing with established eating behavior measures (MEQ, MES), general mindfulness scales (MAAS), and external criteria (dietary intake, BMI) represents a significant methodological limitation. Moreover, demographic data collection was limited to age and gender, with broader sociodemographic characteristics (education level, socioeconomic status) not systematically assessed. Additionally, the use of snowball sampling method in Istanbul may limit generalizability to other Turkish regions with different sociodemographic characteristics. Future validation studies should incorporate these measures alongside known-groups comparisons to provide comprehensive construct validity evidence. These limitations characterize our findings as providing preliminary validity evidence requiring comprehensive construct validation before widespread clinical application.

## 5. Conclusions

The Turkish version of the 17-item MEBS demonstrated excellent psychometric properties (Cronbach’s *α* = 0.855, test-retest = 0.821) and replicated the original scale’s four-factor structure, confirming its validity and reliability for assessing mindful eating behaviors in Turkish adults. The MEBS provides a valuable instrument that measures mindful eating independently of emotional or external eating behaviors, enabling precise assessment of attention-based eating awareness. The validated Turkish MEBS serves as an important tool for developing mindful eating interventions aimed at addressing eating disorders and obesity in the Turkish population. This cross-cultural validation demonstrates the robustness of the MEBS construct and establishes preliminary validity for the scale in Turkish populations. Clinical application requires further construct validity evidence, including convergent and discriminant validity testing with related measures. The Turkish MEBS may contribute to research examining mechanisms related to the eating behaviors underlying obesity in similar populations and enable more targeted interventions for eating behavior problems.

## Figures and Tables

**Table 1:** Distribution of Mindful Eating Behavior Scale items according to factors as a result of EFA.

Items	Factor 1	Factor 2	Factor 3	Factor 4	*h* ^2^	*u* ^2^
1. I notice flavors and textures when I’m eating my food	0.78				0.61	0.39
2. I stay aware of my food while eating	0.53				0.28	0.72
3. I notice how my food looks	0.73				0.53	0.47
4. I notice the smells and aromas of food	0.83				0.69	0.31
5. It is easy for me to concentrate on what I eat	0.50				0.25	0.75
6. I trust my body to tell me when to eat		0.84			0.71	0.29
7. I trust my body to tell me what to eat		0.79			0.62	0.38
8. I trust my body to tell me how much to eat		0.80			0.64	0.36
9. I rely on my hunger signals to tell me when to eat		0.76			0.58	0.42
10. I rely on my fullness signals to tell me when to stop eating		0.58			0.34	0.66
11. I snack without being aware that I am eating			0.73		0.53	0.47
12. I eat automatically without being aware of what I eat			0.85		0.72	0.28
13. I eat something without really being aware of it			0.76		0.58	0.42
14. My thoughts tend to wander while I am eating				0.70	0.49	0.51
15. I think about things I need to do while I am eating				0.81	0.66	0.34
16. I multi-task while I am eating				0.80	0.64	0.36
17. I read while I am eating				0.76	0.58	0.42
Eigenvalue Coefficient	3.29	2.62	2.54	2.43		
Explained Variance	19.35	15.43	14.93	14.30		
Explained Variance (Cumulative)	19.35	34.78	49.71	64.01		

Note: Only loadings ≥ 0.30 are shown; Factor 1 = Focused Eating; Factor 2 = Hunger and Satiety Cues; Factor 3 = Eating with Awareness; Factor 4 = Eating without Distraction. *h*^2^ = communalities; *u*^2^ = uniqueness.

**Table 2:** Goodness-of-fit test values for CFA path diagram of the MEBS (*n* = 397, ML estimation).

Fit Indices	Acceptable Fit Index Criterion	MEBS Fit Indices
*χ* ^2^ */* *df*	3–5	3.147
Goodness of Fit Index (GFI)	0.90–0.95	0.910
Normed Fit Index (NFI)	0.90–0.95	0.905
Comparative Fit Index (CFI)	0.90–0.95	0.918
Root Mean Square Error of Approximation (RMSEA)	0.05–0.08	0.074
Standardized Root Mean Square Residual (SRMR)	<0.08	0.069
Tucker–Lewis Index (TLI)	>0.90	0.887

**Table 3:** Descriptive statistics and reliability indices for MEBS subscales.

Subscale	Mean ± SD	*α* (95% CI)	*ω*	Item-Total *r*
Focused Eating (F1)	4.26 ± 0.52	0.726 (0.67–0.78)	0.788	0.382–0.617
Hunger/Satiety Cues (F2)	3.57 ± 0.89	0.865 (0.84–0.89)	0.871	0.597–0.795
Eating with Awareness (F3)	3.80 ± 0.93	0.821 (0.78–0.86)	0.834	0.608–0.770
Eating without Distraction (F4)	3.37 ± 0.72	0.723 (0.66–0.78)	0.847	0.295–0.530

Note: *α* = Cronbach’s alpha; *ω* = McDonald’s omega; *r* = item-total correlation range within subscale.

**Table 4:** CFA standardized factor loadings and model parameters.

Item	Factor	Standardized Factor Loadings (*λ*)	Residual	*R* ^2^
Item 1	F1	0.742	0.450	0.551
Item 2	F1	0.521	0.729	0.271
Item 3	F1	0.695	0.517	0.483
Item 4	F1	0.798	0.363	0.637
Item 5	F1	0.485	0.765	0.235
Item 6	F2	0.824	0.321	0.679
Item 7	F2	0.798	0.363	0.637
Item 8	F2	0.812	0.341	0.659
Item 9	F2	0.743	0.448	0.552
Item 10	F2	0.601	0.639	0.361
Item 11	F3	0.756	0.429	0.571
Item 12	F3	0.834	0.304	0.696
Item 13	F3	0.782	0.389	0.611
Item 14	F4	0.721	0.480	0.520
Item 15	F4	0.798	0.363	0.637
Item 16	F4	0.785	0.384	0.616
Item 17	F4	0.743	0.448	0.552

Note. All factor loadings are significant at *p* < 0.001. Factor correlations: F1–F2 = 0.290; F1–F3 = 0.293; F1–F4 = 0.200; F2–F3 = 0.562; F2–F4 = 0.340; F3–F4 = 0.454.

## Data Availability

The anonymized aggregated datasets and statistical analysis syntax used in this study are available from the corresponding author upon reasonable request for research reproducibility purposes. Data sharing is subject to participant privacy protection and ethics board requirements. Individual participant data cannot be shared to ensure confidentiality, as per the ethical approval conditions.

## Abbreviations

MEBS, Mindful Eating Behavior Scale; EFA, Exploratory Factor Analysis; CFA, Confirmatory Factor Analysis; KMO, Kaiser–Meyer–Olkin; BMI, Body Mass Index; MEQ, Mindful Eating Questionnaire; MES, Mindful Eating Scale; GFI, Goodness of Fit Index; CFI, Comparative Fit Index; NFI, Normed Fit Index; RMSEA, Root Mean Square Error of Approximation; CVI, Content Validity Index; I-CVI, Item-Level Content Validity Index; S-CVI, Scale-Level Content Validity Index
